# Supplementary material for: De Novo Assembly and Annotation of the Vaginal Metatranscriptome Associated with Bacterial Vaginosis
Source: Int J Mol Sci. 2022 Jan 30;23(3):1621. doi: 10.3390/ijms23031621 (PMC8835865; doi:10.3390/ijms23031621)
Supplement: Supplementary file 1 [file ijms-23-01621-s001.zip › Supplementary Figures.pdf]

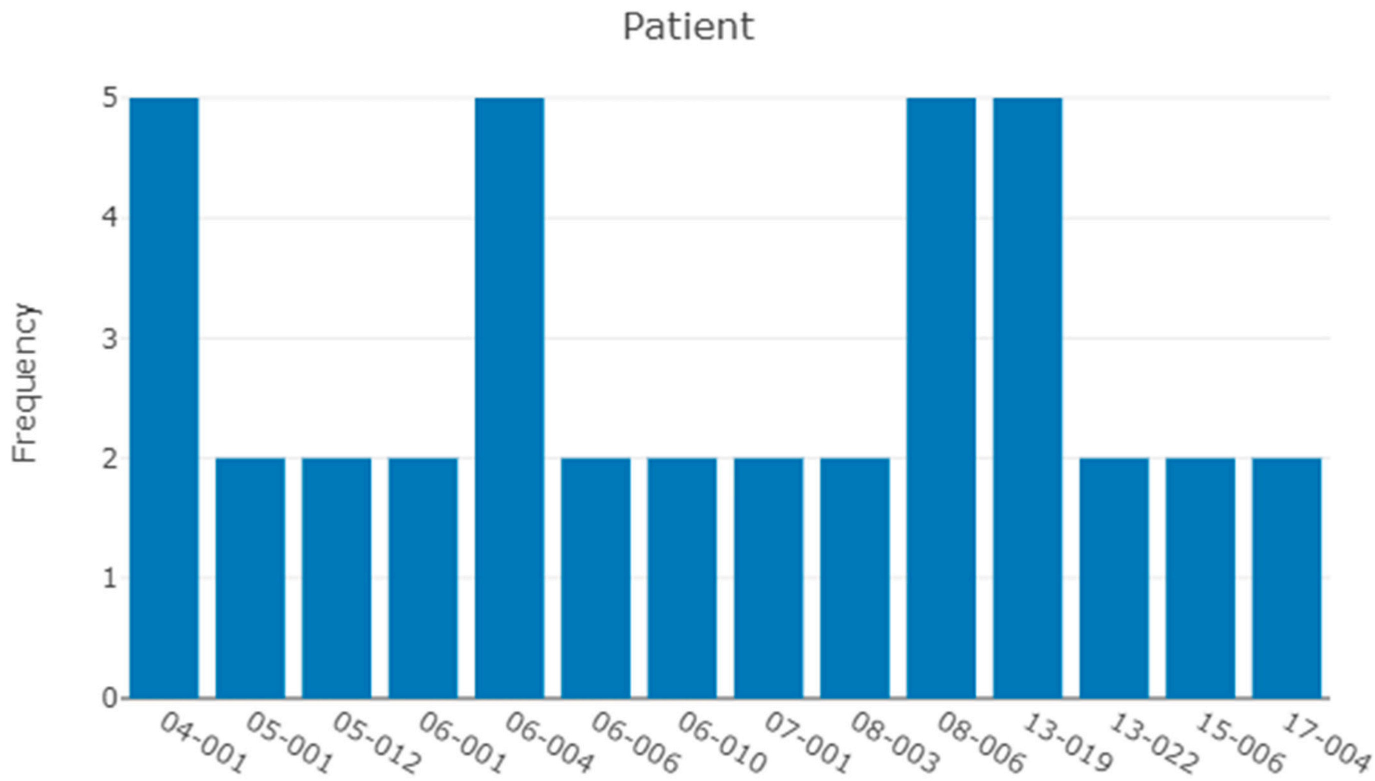

Figure S1. Number of vaginal samples collected from each of 14 patients.

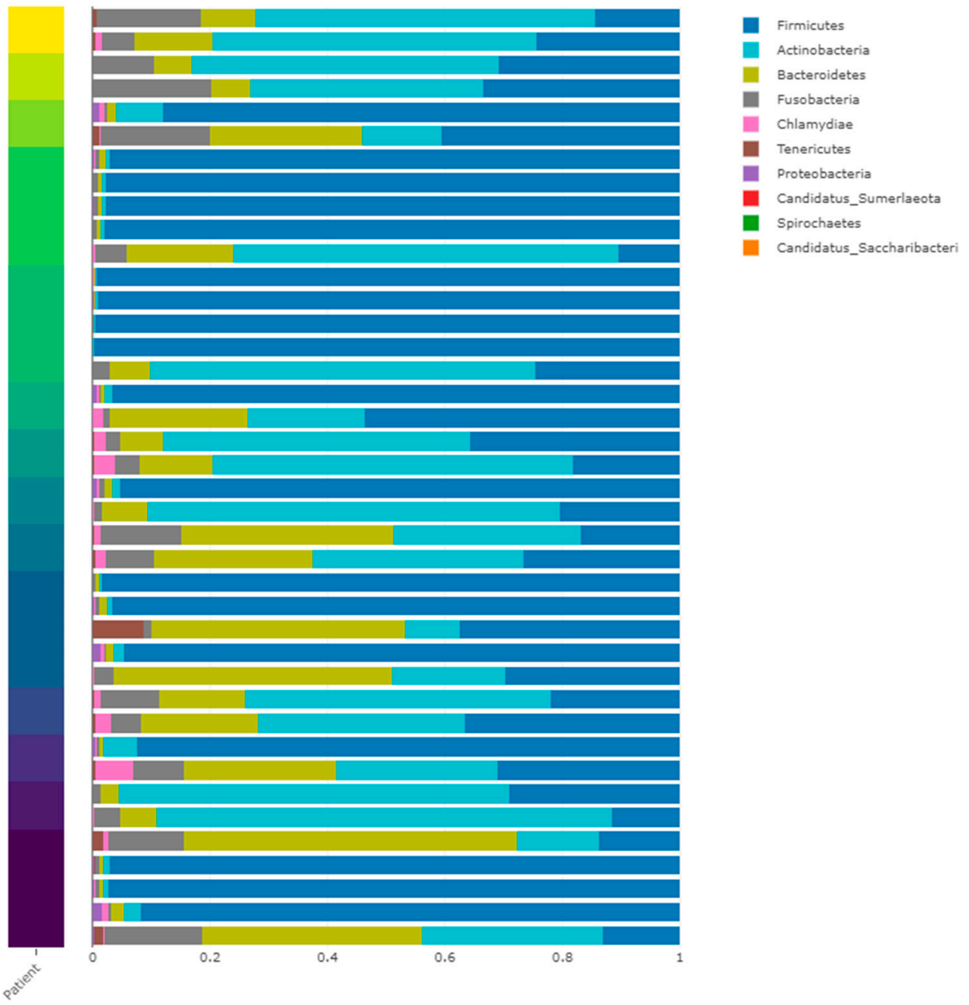

Figure S2. Relative abundance of identified bacteria in 40 different samples at phylum level.

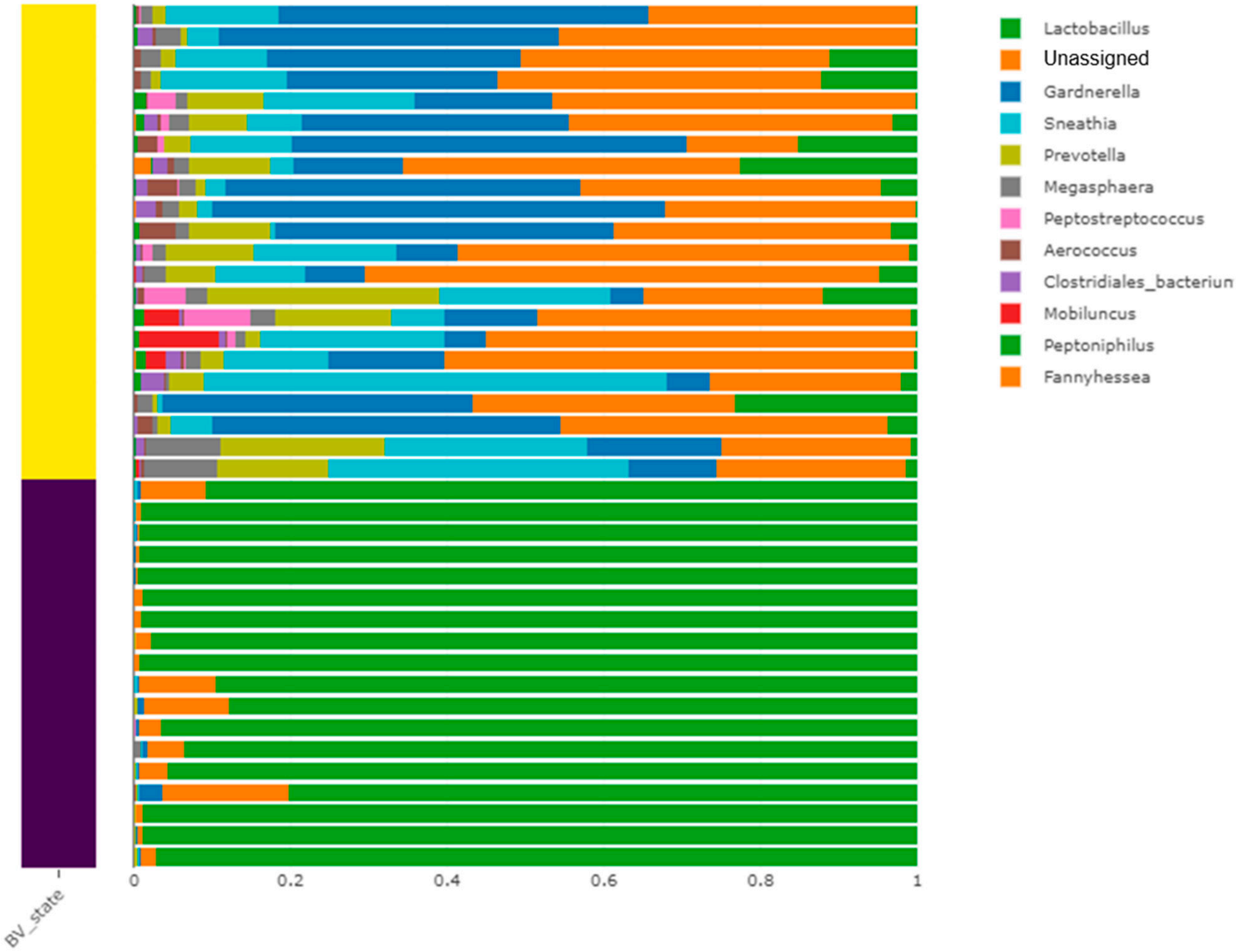

Figure S3. Relative abundance of 176 bins in 40 different samples at genus level. Yellow and purple bars indicate positive and negative samples for BV state, respectively.

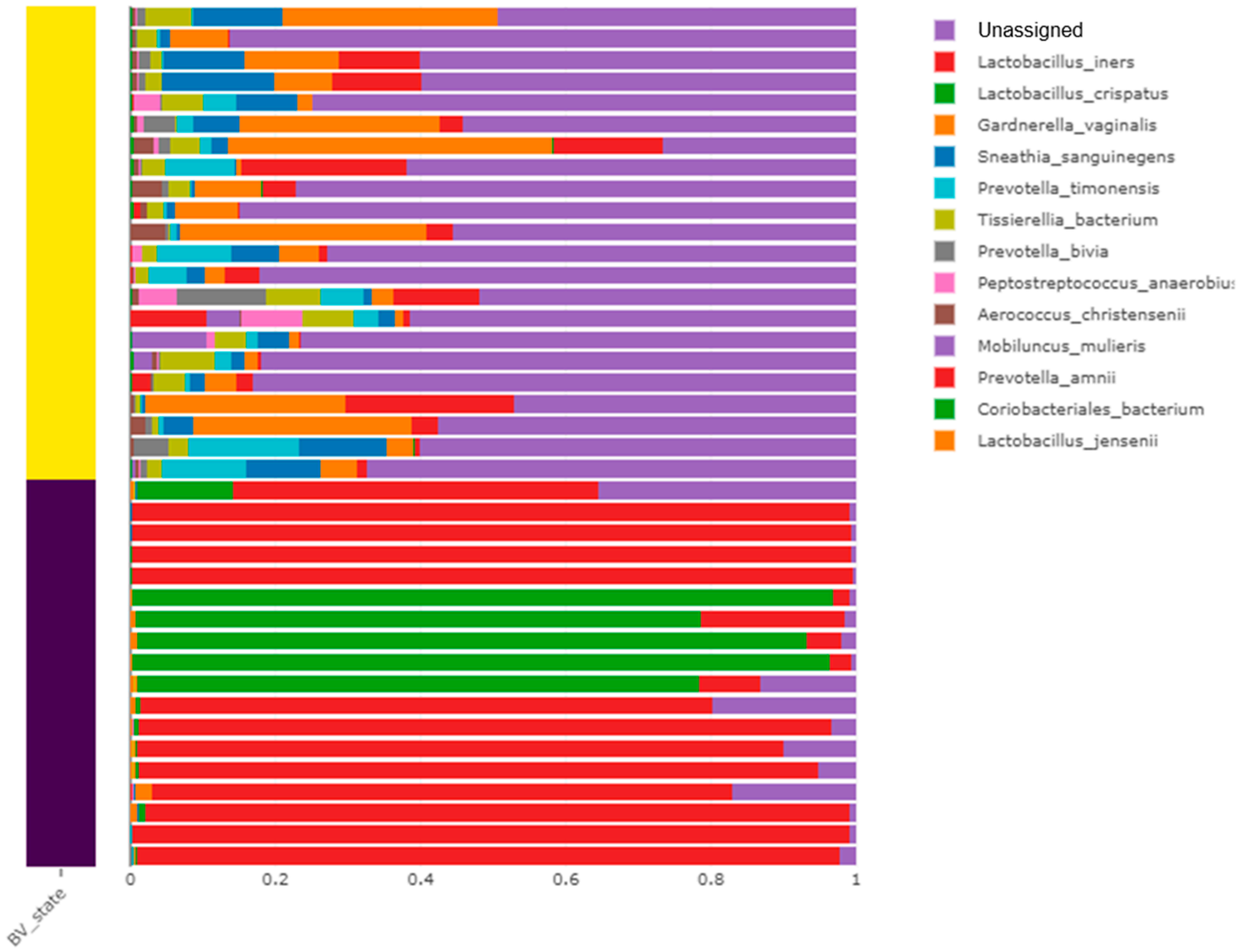

Figure S4. Relative abundance of 176 bins in 40 different samples at species level. Yellow and purple bars indicate positive and negative samples for BV state, respectively.

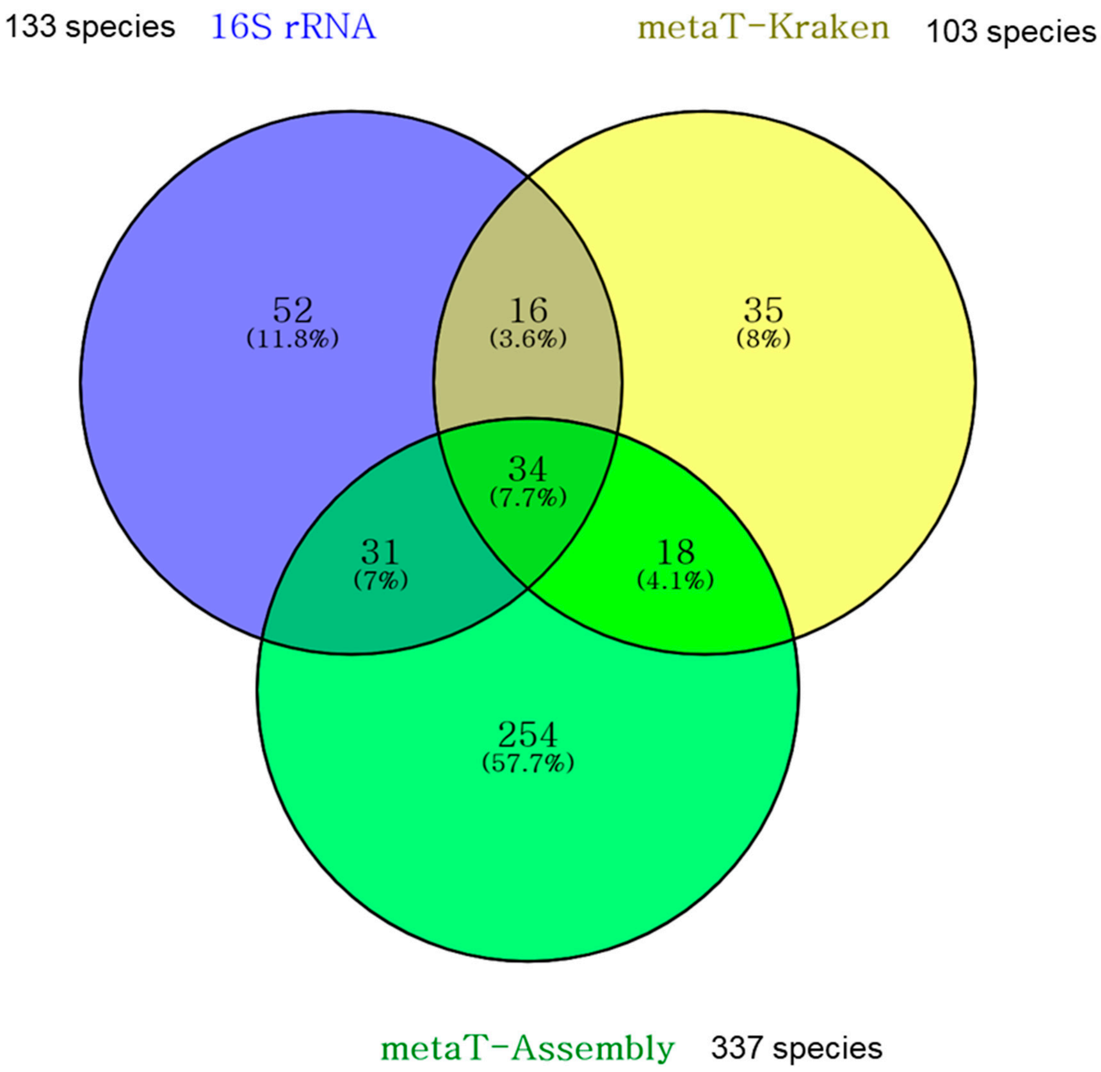

Figure S5. Comparison of number of identified bacterial species from three different studies.

16S rRNA indicates the previous study using 16S rRNA amplicon sequencing from 96 samples. metaT-Kraken indicates the previous study using metatranscriptomic data from 40 samples using Kraken. metaT-Assembly indicates this study using metatranscriptomic data from 40 samples using de novo transcriptome assembly followed by BLASTX search against an NR protein database.
